# Homocysteine, Vitamins B6 and Folic Acid in Experimental Models of Myocardial Infarction and Heart Failure—How Strong Is That Link?

**DOI:** 10.3390/biom12040536

**Published:** 2022-04-01

**Authors:** Zorislava Bajic, Tanja Sobot, Ranko Skrbic, Milos P. Stojiljkovic, Nenad Ponorac, Amela Matavulj, Dragan M. Djuric

**Affiliations:** 1 Department of Physiology, Faculty of Medicine, University of Banja Luka, 78000 Banja Luka, Bosnia and Herzegovina; zorislava.bajic@med.unibl.org (Z.B.); tanja.sobot@med.unibl.org (T.S.); nenad.ponorac@med.unibl.org (N.P.); amela.matavulj@med.unibl.org (A.M.); 2 Department of Pharmacology, Toxicology and Clinical Pharmacology, Faculty of Medicine, University of Banja Luka, 78000 Banja Luka, Bosnia and Herzegovina; ranko.skrbic@med.unibl.org (R.S.); milos.stojiljkovic@med.unibl.org (M.P.S.); 3 Faculty of Medicine, Institute of Medical Physiology “Richard Burian”, University of Belgrade, 11000 Belgrade, Serbia

**Keywords:** homocysteine, vitamin B6, folic acid, myocardial infarction, heart failure, experimental models

## Abstract

Cardiovascular diseases are the leading cause of death and the main cause of disability. In the last decade, homocysteine has been found to be a risk factor or a marker for cardiovascular diseases, including myocardial infarction (MI) and heart failure (HF). There are indications that vitamin B6 plays a significant role in the process of transsulfuration in homocysteine metabolism, specifically, in a part of the reaction in which homocysteine transfers a sulfhydryl group to serine to form α-ketobutyrate and cysteine. Therefore, an elevated homocysteine concentration (hyperhomocysteinemia) could be a consequence of vitamin B6 and/or folate deficiency. Hyperhomocysteinemia in turn could damage the endothelium and the blood vessel wall and induce worsening of atherosclerotic process, having a negative impact on the mechanisms underlying MI and HF, such as oxidative stress, inflammation, and altered function of gasotransmitters. Given the importance of the vitamin B6 in homocysteine metabolism, in this paper, we review its role in reducing oxidative stress and inflammation, influencing the functions of gasotransmitters, and improving vasodilatation and coronary flow in animal models of MI and HF.

## 1. Introduction

Cardiovascular diseases (CVD), particularly ischemic heart and brain diseases, are the leading cause of death and the main cause of disability today. The results of the Global Burden of Disease Study showed that in the period from 1990 to 2019, the prevalence of total CVD almost doubled, from 271 million to 523 million. The number of deaths from these diseases also increased, from 12.1 million to 18.6 million [1], with an estimate of reaching a staggering 23 million by 2030 [2]. Additionally, the disability-adjusted life years (DALYs) due to the ischemic heart disease reached 182 million DALYs. The burden of CVD is growing in almost all countries, and the prevalence of CVD is also rising in some highly developed countries in which a decline has previously been reported [1].

The main cardiovascular risk factors include family history of CVD, smoking, sedentary lifestyle, obesity, alcohol use, diabetes, dyslipidemia, and hypertension [3,4]. It is considered that all of these factors promote low-density lipoprotein (LDL) uptake into the vascular wall [4], causing lipid accumulation in the subendothelial layer and inducing atherogenesis. Atherogenesis is responsible for the activation of endothelial cells by monocytes and the formation of foam cells. This leads to accumulated smooth muscle cells in artery walls that can form a fibrous atheroma cap, thereby contributing to atherosclerosis [3]. High blood pressure (hypertension) and high blood cholesterol can contribute to the exacerbation of atherosclerosis through several mechanisms, such as the renin–angiotensin–aldosterone system (RAAS), impaired endothelial function, and oxidative stress. Hypertension is also associated with a compromised regulation of methionine cycle, leading to the elevated level of homocysteine [3,4,5]. Elevated homocysteine concentration could damage the endothelium of blood vessels, thus participating in pathophysiology of CVD [5,6].

## 2. Metabolism of Homocysteine

### 2.1. Homocysteine Methabolism Pathaway

**Figure 1:**
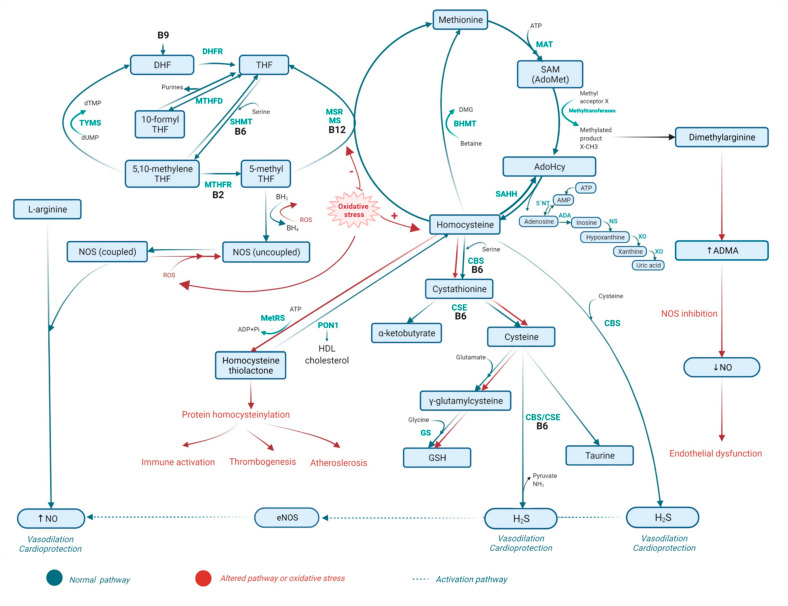
Homocysteine metabolism and folate cycle. ADA—adenosine deaminase, ADMA—asymmetric dimethylarginine, AdoMet—S-adenosyl methionine, AdoHcy—S-adenosyl homocysteine, B2—vitamin B2, B6—vitamin B6, B9—vitamin B9, B12—vitamin B12, BH2—dihydropterin, BH4—tetrahydrobiopterin, BHMT—betaine homocysteine methyltransferase, CBS—cystathionine β-synthase, CSE—cystathionine γ-lyase, DHF—dihydrofolate, DHFR—dihydrofolate reductase, DMG—dimethylglycine, dTMP—deoxythymidine monophosphate, dUMP—deoxyuridine monophosphate, eNOS—endothelial NOS, GS—glutathione synthase, GSH—reduced glutathione, H_2_S—hydrogen sulfide, MAT—methionine adenosyltransferase, MetRS—methionyl-tRNA synthetase, MS—methionine synthase, MSR—methionine synthase reductase, MTHFD—methylenetetrahydrofolate dehydrogenase, MTHFR—methylenetetrahydrofolate reductase, NO—nitric oxide, NOS—nitric oxide synthase, NS—nucleosidase, PON 1—paraoxonase 1, ROS—reactive oxygen species, SAHH—S-adenosyl homocysteine hydrolase, SAM—S-adenosyl methionine, SHMT—serine hydroxymethyltransferase, THF—tetrahydrofolate, TYMS—thymidylate synthase, XO—Xanthine oxidase, 5′NT—nucleotidase. The ability of H_2_S to increase NO bioavailability by activating eNOS is depicted with the dashed arrow. Arrows pointing up (↑) next to substance or enzyme represent increased level of substance or increased enzyme acitvity, and arrows pointing down (↓) represent decreased level of substance or decreased enzyme activity.

Homocysteine (Hcy) is considered to be a marker of CVD and is known as a risk factor, in particular, for stroke, myocardial infarction (MI), heart failure (HF), cancer, Alzheimer’s disease, and atherosclerosis [7,8,9]. Hcy is a metabolite of methionine, meaning that Hcy is also included in the folate cycle. Hcy can be formed from methionine through the process of resynthesis, or from cysteine by its degradation [10]. Hcy does not enter the body with food. Rather, it is produced from methionine in a complex process involving many steps [7,10], as follows (see also Figure 1). In a first step, the adenosine group is converted from ATP to methionine with the help of the enzyme S-adenosyl methionine (SAM or AdoMet), which is a synthetase also referred to as methionine adenosyltransferase (MAT). In a subsequent step, SAM delivers the methyl group to recipient molecules, such as proteins, RNA, DNA, and neurotransmitters. This process is known as one-carbon metabolism. More than 100 different methyltransferases are involved in this process, resulting in the formation of the S-adenosyl Hcy (AdoHcy), which acts as an inhibitor of most methyltransferases [7,10].

In the next step, l-homocysteine is formed by means of AdoHcy hydrolase (SAHH). The reaction here goes in both directions, although it is predominantly in the direction of AdoHyc synthesis [7,10]. Additionally, adenosine can result from this reaction, but can also result from AMP via 5′-nucleotidase. In adenosine metabolism, adenosine forms inosine by adenosine deaminase (ADA). From here, inosine produces hypoxanthine by means of nucleosidase. Thereafter, hypoxanthine forms xanthine and uric acid via xanthine oxidase [3]. Hcy and adenosine should be expelled from the cell to prevent their accumulation [7,10]. Adenosine can modulate inflammation and immune response. Accumulation of adenosine is associated with CVD, especially in myocardial ischemia [3]. It is also worth noting that excessive accumulation of AdoHcy is harmful, since it can lead to general DNA hypomethylation [7].

Newly formed L-homocysteine can now undergo two processes: (i) remethylation, where l-methionine is formed as the product, and (ii) transsulfuration, where L-cysteine is formed as the product. *Remethylation* is a process in which Hcy is transformed to methionine. This reaction involves methionine synthase (MS), an enzyme that links homocysteine to folate metabolism [11]. The cofactor cobalamin (Cbl or vitamin B12) is necessary for the action of this enzyme. Together, they form the Cbl (I) MS complex, which in its turn binds the methyl group of 5-methyl tetrahydrofolate (5-MTHF). 5-MTHF then serves as a donor of methyl group. Oxidation of cobalamin forms an inactive Cbl (II) MS complex. Methionine synthase reductase (MSR) reactivates the Cbl (II) MS complex by reduced methylation, where AdoMet serves as a methyl donor [10]. The result of this remethylation pathway is the formation of methionine and tetrahydrofolate (THF). As enzyme MS is present in all parts of the human organism, this type of remethylation can take place anywhere in the body. Furthermore, the remethylation process can occur in the liver and kidneys with the enzyme called betaine-homocysteine methyltransferase (BHMT). In this case, betaine (trimethyl glycine) acts as a methyl group donor. A cofactor of this enzyme is zinc, and the products of these reactions are dimethylglycine (DMG) and methionine [7].

*Transsulfuration* is the process during which homocysteine forms cysteine. This process includes two enzymes, cystathionine β-synthase (CBS) and serine. The catalyst of the reaction is cystathionine γ-lyase (CSE), which hydrolyses cystathionine to cysteine and α-ketobutyrate. These are the two key enzymes in the production of hydrogen sulfide (H_2_S) from cysteine. H_2_S can also be a result of the reaction of homocysteine and cysteine, catalyzed by CBS [12]. Vitamin B6 (priridoxal-5′-phosphate) is required for the action of both enzymes, CBS and CSE [7,10,13,14,15]. Cysteine produced in the process of transsulfuration can be used for synthesis of glutathione (GSH) and proteins [16]. As already mentioned, B vitamins (B6, B12, B2) are necessary for the normal metabolism of Hcy. Deficiency of these vitamins can cause accumulation of Hcy and can lead to toxic effects including cell and tissue damage [7].

The abovementioned toxic effects of Hcy are a result of the following: inhibition of Na^+^, K^+^ ATPase, oxidative stress, inhibition of acetylcholinesterase, inhibition of production of gasotransmitters (NO, CO, H_2_S), overstimulation of n-methyl-D-aspartate (NMDA) receptors, and inhibition of cardiac tissue respiration [13]. Hcy can appear in several forms, including as mixed homocysteine disulfides and s-homocysteinylated proteins. The reduced form of homocysteine accounts for less than 1% of total Hcy. Oxidized forms (s-homocysteinylated proteins and n-homocysteinylated proteins) account for approximately 80% of total Hcy. The precursor of n-homocysteinylated proteins is homocysteine thiolactone (HTL) [17]. In physiological conditions, the concentration of HTL is low. However, hyperhomocysteinemia (HHcy) is characterized by a larger amount of HTL. The reaction leading to HHcy is catalyzed by methionyl-tRNA synthetase (MetRS), as depicted in Figure 1 [13,18]. In vitro studies in human cells and serum have shown that HTL undergoes two reactions: (i) homocysteinylation of a protein on a lysine residue, and (ii) enzymatic hydrolysis to Hcy by a calcium-dependent hydrolytic enzyme called paraoxonase 1 (PON1) [18,19].

PON1 is produced in the liver, and in the circulation it binds high-density lipoprotein (HDL) [20], contributing to anti-inflammatory, antioxidative, and antiatherothrombotic effects of HDL [18,19]. Animal and human studies have shown that PON1 has a protective effect against atherosclerosis induced by a high-fat diet [21,22].

### 2.2. Folate Metabolism

Folate metabolism is closely related to Hcy metabolism (Figure 1) [7,10,23]. It acts as a methyl donor in the homocysteine remethylation process [10]. Folate is one of the B complex vitamins, and it is essential for the functioning of specific enzymes. The primary metabolite of folate is 5-MTHF [24]. 5-MTHF enters the blood circulation from intestinal cells and is transported via blood to specific cells. The conversion of folate to 5-MTHF is limited. This enables organism to deal with ingestion of substantial amounts of folates by not converting them all, but instead circulating them in non-metabolized form. In this case, it enters the cell, and dihydrofolate (DHF) is reduced to THF by dihydrofolate reductase (DHFR) [24]. The enzyme serine hydroxymethyltransferase (SHMT) can directly convert THF to 5,10-methylene THF. SHMT uses serin as a carbon donor, and requires vitamin B6 for its normal function [10].

In the cycle of folate, when 5,10-methylene THF acts as a co-substrate for the conversion of deoxyuridine monophosphate (dUMP) to deoxythymidine monophosphate (dTMP), 10-formyl THF gives a carbon group for purines biosynthesis (Figure 1). Enzyme thymidylate synthase (TYMS) participates in conversion of dUMP to dTMP. This process produces dihydrofolate (DHF). Dihydrofolate reductase reduces DHF to THF. In addition to being a cofactor in the formation of dTMP, 5,10-methylene THF is reduced to 5-MTHF by the methylenetetrahydrofolate reductase (MTHFR). The normal function of MTHFR is dependent on riboflavin (vitamin B2). However, TYMS and MTHFR compete for 5,10-methylene THF. That makes MTHFR the major enzyme for controlling 5-methyl THF production in the process of homocysteine remethylation [7,10]. 5-methyl THF is an active form of folate circulating in plasma. It is transported to the target cells of peripheral tissues and is used in cellular metabolism [25]. Folate deficiency leads to hyperhomocysteinemia [7,10].

### 2.3. The Role of Vitamins B6 and B12 in Homocysteine Metabolism and the Folate Cycle

Vitamins B6 and B12 have very important functions in one-carbon metabolism, and through that they are linked to the metabolism of Hcy [26,27]. Vitamin B6 is important in the process of transsulfuration of Hcy metabolism. Namely, vitamin B6 participates in the reaction in which Hcy transfers a sulfhydryl group to serine to form α-ketobutyrate and cysteine (Figure 1). Cysteine is a precursor of the main antioxidant component, glutathione (GSH). In the process of transsulfuration, vitamin B6 is a cofactor necessary for the normal function of the enzymes CBS andCSE. An insufficient amount of methionine induces a homocysteine remethylation reaction, resulting in methionine synthesis. This reaction requires 5-methyltetrahydrofolate as a substrate and vitamin B12 as a cofactor for MS [28]. Another important role of vitamin B12 is its role as a cofactor for SHMT, which converts THF to 5,10-methylene THF [10].

### 2.4. Metabolic Disorders Related to Altered Homocysteine Metabolism

Hyperhomocysteinemia (HHcy), increased concentration of Hcy in plasma, can be classified as mild (15–30 μmol/L), medium (30–100 μmol/L) or severe (more than 100 μmol/L). The causes of HHcy fall into the following five categories: (i) enzyme disorder, (ii) cofactor deficiency, (iii) excessive methionine intake, (iv) specific diseases such as chronic renal failure, hypothyroidism, anemia, or malignant tumors, and (v) intake of specific drugs such as cholestyramine, methotrexate, oral contraceptive pills, phenytoin, carbamazepine, or metformin [7,29]. These five categories of HHcy causes are discussed in detail in the following paragraphs.

As indicated above, one of the common causes of HHcy in Hcy metabolism is enzyme disorders, i.e., deficiency or genetic effects. For example, deficiency of the enzyme CBS may lead to elevated Hcy concentrations due to its pivotal role in the conversion of Hcy to cystathionine. When this enzyme does not perform its function in the transsulfuration process, an insufficient amount of Hcy converts into cystathionine. Additionally, genetic defects of the enzymes MTHFR and MS pose a risk of HHcy [7].

HHcy can be also caused by the deficiency of cofactors involved in the metabolism of Hcy, namely vitamins B2, B6 and B12 [30]. These vitamins are water soluble and therefore easily excreted in the urine. Deficiency of vitamins B12 and B2, which participate in the remethylation process, and deficiency of vitamin B6, as a cofactor in the process of transsulfuration, often occurs in the elderly population. As a consequence, HHcy is more common in the ageing population [31]. There is a 2.5-fold increase in HHcy with folate deficiency, and a 2.6-fold increase with vitamin B12 deficiency [32].

Methionine, as an essential amino acid, is the only source of Hcy in food. A methionine-rich diet stimulates Hcy production. Mice fed with the methionine or Hcy-rich diets had higher concentrations of homocysteine-thiolactone in the urine compared to mice fed with a balanced diet. Plasma homocysteine-thiolactone concentrations were also elevated, although this difference was not significant [33].

Renal failure, hypothyroidism, anemia, and malignant tumors are all associated with HHcy. In renal failure, HHcy can be caused by reduced glomerular filtration and renal excretion [34]. In addition, the kidney tissue can contain the enzymes involved in the process of remethylation and transsulfuration. However, in chronic renal failure, these enzymes are inactive. HHcy in renal failure can also occur due to non-renal causes such as impaired folate metabolism [35].

Elevated Hcy concentrations are present in patients with clinical and subclinical forms of hypothyroidism [36,37]. There are no consistent data on the HHcy mechanism for this condition. There is a correlation between elevated concentrations of Hcy and Hashimoto thyroiditis (the most common cause of hypothyroidism). Patients with iatrogenic hypothyroidism have higher Hcy concentrations compared to those without hypothyroidism. This suggests that immunological inflammatory diseases can increase Hcy levels [38]. Vitamin B12 and folate deficiency can cause megaloblastic anemia. These vitamins are necessary for normal Hcy metabolism, and their deficiency can lead to HHcy [7].

Furthermore, Vitamin B12 and folate deficiency can also be found in various neoplasms. Tumors impair normal Hcy metabolism mostly due to folate deficiency [39,40]. Tumor cells release substantial amounts of Hcy. Rapidly proliferating tumor cells use folate for their metabolism and inactivate the remethylation process catalyzed by MS, causing HHcy [41].

HHcy is associated with DNA damage and neurotoxicity by increased n-methyl-D-aspartate (NMDA) receptor expression [42,43]. HHcy also alters the bone microarchitecture and increases the risk of bone fracture [44]. An experimental study showed that Hcy can reduce the activity of cardiac acetylcholinesterase and alter heart function [45].

### 2.5. Consequences of Homocysteine, Folate, and Vitamin B6 Metabolism-Related Disorders

The mechanism by which Hcy causes or participates in the onset of certain diseases has not yet been fully understood on the basis of the current literature. Elevated Hcy levels have been found in different pathological conditions, such as CVD, diabetes, vision and hearing impairment, cognitive dysfunction, hypertension, carcinomas, and bone fractures.

The relationship between Hcy and CVD requires special attention. Namely, it is considered that HHcy can cause damage to endothelial function [7,13]. Endothelial dysfunction is the basis for the development of CVDs [46]. Therefore, in this section, the focus will be on the Hcy, folic acid, and vitamin B6 metabolism disorders in relation to CVDs.

#### 2.5.1. Homocysteine and CVD

Elevated Hcy concentration affects blood vessels and could be responsible for the worsening of atherosclerosis by means of damaging the blood vessel wall [6]. Endothelial cells are sensitive even to moderate concentrations of Hcy. Elevated Hcy concentration can alter vascular endothelial functions—its surface changes its characteristics from an anticoagulant to a procoagulant state [47]. Hcy stimulates the production of the platelet thromboxane A2 [48]. HHcy activates coagulation factor V, resulting in the impairment of protein C activation and thrombomodulin expression. Elevated Hcy concentration reduces the efficacy of anticoagulant substances and inhibits their DNA synthesis [49]. Hcy and its thiolactone form inhibit the activity of lysyl oxidase (an enzyme that participates in the maturation of the extracellular matrix) in the cells of the vascular endothelium [50]. Hcy and Hcy compounds also induce depression of heart contractility and coronary flow [51,52] and decrease oxygen consumption [53]. In vitro study has shown that incubation of isolated rat femoral artery with Hcy damages the vascular endothelium via discrete or expressed interruption of endothelial cells [54]. The key pathophysiological mechanism of Hcy-induced atherosclerosis is its ability to stimulate production of reactive oxygen species (ROS). Auto-oxidized sulfhydryl groups of Hcy stimulate the production of ROS leading, in turn, to deactivation of endothelial nitrogen monoxide (NO). High concentrations of Hcy disturb the activity of glutamate-cysteine ligase (previously known as gamma-glutamylcysteine synthetase). This enzyme is responsible for de novo glutathione synthesis [55]. Consequently, the concentration of glutathione decreases, leading to a reduction in the detoxification of ROS. As glutathione reduces ROS production and stimulates NO production, reduced concentration of glutathione can, therefore, directly and indirectly cause the hypercoagulable state on the endothelial surface [56]. HHcy stimulates the proliferation of smooth muscle cells in the blood vessels intima (and they increase collagen synthesis). This leads to an irregular extracellular matrix formation, which affects the appearance and worsening of atherosclerosis [57,58]. Smooth muscle cells increase collagen synthesis in the presence of Hcy. High Hcy concentrations cause a condition similar to inflammation due to an interaction between endothelial cells and neutrophils, as well as neutrophil migration [57]. Experimental studies have shown that a high-methionine diet induces HHcy, leading to impaired myocyte contractility, increased cardiac remodeling, and procoagulant properties in the endothelium [59,60,61,62,63]. These studies also showed that statins attenuate Hcy-induced myocyte contractility impairment [62,63]. Elevated levels of Hcy also lead to hyperuricemia. Uric acid increases the amount of ROS, which causes a decrease in the bioavailability of NO and results in endothelial dysfunction [64].

Endothelial dysfunction plays an important role in all stages of coronary artery disease (CAD) [65]. All of the abovementioned mechanisms involved in endothelial dysfunction also participate in the development of atherosclerotic CAD [66]. The role of folic acid and vitamins B6 and B12 in the metabolism of Hcy is already known [7], and the literature confirms that the consumption of B-group vitamins reduces Hcy level in the circulation [67]. These vitamins help in the treatment and/or prevention of Hcy metabolism disorders. The results of a study investigating the primary prevention of CAD by folic acid supplementation showed an inverse relationship between high doses of folic acid (200 µmol/day) and the incidence of CAD in follow-up period of 10.7 years [68]. The primary prevention and protective effects of folic acid and vitamins B6 and B12 were the focus of a large Japanese study in which a significant inverse relationship of the highest and the lowest folate levels with CAD mortality in women was found [69]. Several studies have shown that folic acid and vitamin B12 supplementation can lower Hcy concentration [70], and supplementation with folic acid can improve damaged vascular endothelial function in CAD [71]. HHcy is also positively correlated with the severity of CAD [72]. Another study showed a positive correlation between Hcy level and CAD, as well as significant negative correlation between Hcy levels and folic acid concentration in all CAD categories [66]. Another study [73] found that AMI patients had lower levels of pyridoxal phosphate (PLP) and folic acid compared to healthy individuals. Hcy levels were higher than in the control group, although there were also no significant differences [73].

Stroke is another clinical condition associated with atherosclerosis, HHcy, and reduced concentrations of folic acid and B vitamin. The meta-analysis of 19 randomized controlled trials included an analysis of the effects of folic acid and vitamins B6 and B12 on Hcy concentration, primary prevention of CVDs, and mortality. The results showed that the incidence of stroke was significantly reduced by the use of folic acid and B6 and B12 vitamins [74]. A large randomized double-blind study, the China Stroke Primary Prevention Trial (CSPPT), focused on the primary prevention of stroke in subjects with hypertension. The resuls showed that the risk of the first ischemic stroke and other cardiovascular events, such as MI and cardiovascular death, was lower in the group co-treated with antihypertensive therapy and folic acid [75]. A meta-analysis of three large randomized control studies—Vitamin Intervention for Stroke Prevention (VISP), Vitamins to Prevent Stroke (VITATOPS), and Heart Outcomes Prevention Evaluation 2 (HOPE-2)—focused on the protective effects of folic acid plus B vitamins [76]. The results showed a lower incidence of recurrent stroke in a group of patients receiving folic acid and vitamins B6 and B12 [77,78,79,80]. Another meta-analysis showed that treatment with folic acid alone or in combination with vitamin B12 was associated with a lower risk of recurrent stroke [81].

HHcy due to reduced concentration of vitamins B6, B12 and folic acid is considered a part of the venous thrombosis pathophysiological mechanism [82]. A mutation in the MTHFR gene, as well as HHcy, increases the risk of deep vein thrombosis (DVT) [83]. The results of a clinical study [84] showed that 15% of patients with DVT had HHcy, of which 43.3% had decreased folic acid concentration (less than 3 ng/mL), 1.6% had decreased vitamin B12 concentration (less than 150 pmol/L), and 3.33% had decreased vitamin B6 concentration (less than 30 nmol/L). The average Hcy concentration was higher in women compared to men, and a significant correlation was found between Hcy concentration and age [84]. Namely, patients with DVT older than 40 years had higher Hcy concentrations than the younger age group. This difference could be explained by the prevalence of CVD, cognitive disorders, and depression in the elderly (conditions associated with HHcy) [15]. An increase in Hcy concentration by 5 µmol/L increases the risk of all-cause mortality by 27%, the risk of CVD by 32%, and CAD by 52% [85]. This risk increases with age [85]. The available literature also shows that HHcy in women older than 40 years is a risk factor for DVT [84].

Considering that a plethora of CVDs, including CAD, MI, heart valve disease, and hypertension, can lead to heart failure (HF) [86], it is important to analyze the relationship between Hcy and HF. In vitro research on cardiovascular cells and tissue showed the sensitivity of myocardial cells to damage caused by Hcy [87]. Clinical study with chronic HF patients found a correlation between HHcy and increased mortality rate [88]. The mechanism by which Hcy affects HF is unknown, and it is unclear whether HHcy may be a direct cause of HF. Heart diseases in general and HF in particular, are complex, and are influenced by so many factors that it cannot be concluded that Hcy is the only factor that can cause or worsen HF [7]. It can be considered as a possible marker of dyslipidemia, and can therefore be used as a factor influencing the deterioration and progression of CVD [89]. There are also indications that Hcy can be used as a prognostic marker of long-term heart complications in patient with chronic HF [90].

#### 2.5.2. Homocysteine and Diabetes

Diabetes mellitus (DM) can alter Hcy metabolism [91]. Hypomethylated genomic DNA has been found in diabetic rats [92]. Folate is involved in SAM synthesis, which acts as a methyl donor for the proteins DNA and RNA [7]. Clinical studies have shown the beneficial effects of folic acid supplementation in patients with DM, and these include reduced Hcy concentration, reduced fasting insulin, and better control of glycemia [93,94]. Animal studies have also shown the beneficial effects of folic acid in streptozotocin-induced diabetes mellitus in rats. The results of these studies showed hepato- and cardio-protective effects as well as glycemia reduction in diabetic rats treated with folic acid [95,96]. Folic acid also reduces cardiomyocyte apoptosis in experimentally induced diabetic rats [97].

Vitamin B6 has an inverse relationship with DM. Deficiency of vitamin B6 is associated with DM complications. Pyridoxal 5′-phosphate (PLP) concentration is inversely related to complication in DM patients [98]. A study on alloxan-induced diabetic rats showed a significant reduction in blood glucose level, better physical performance, and decreased ROS production and damage of DNA after administration of vitamin B6 [99]. Since oxidative stress plays a significant role in DM, it is considered that a low level of vitamin B6 cannot fulfil its antioxidant function [100].

#### 2.5.3. Homocysteine and Drugs

Lipid-lowering drugs, diabetes drugs, sex hormones, antirheumatic drugs, antiepileptic drugs (and other such as levodopa, acetylcystein, cyclosporin) can alter Hcy metabolism. Namely, lipid-lowering drugs can increase levels of Hcy, but the mechanism by which fibric acid derivatives do that is not well understood. Nicotinic acid can increase Hcy level via inhibition of pyridoxal kinase, decreased vitamin B6 level, and increased CBS activity. Cholestyramine increases Hcy level by interfering with folate and vitamin B12 absorption. Insulin increases MTHFR activity and decreases CBS activity, causing the Hcy level to decrease. Sex hormones can increase or decrease Hcy concentration, estrogens causing a decrease and androgens causing an increase. Methotrexate inhibits activity of DHFR and causes increased Hcy levels. Phenytoin and carbamazepine increase Hcy via folate depletion and possible hepatic enzyme induction, and phenyotine alone also via a possible decrease in 5-MTHFR and methionine synthase activity. Levodopa is a substrate for AdoMet-dependent transmethylation, and it increases Hcy concentration. Acetylcysteine decreases Hcy via thiol-sulfide exchange. Cyclosporine can increase Hcy concentration through possible interference in the remethylation process of Hcy metabolism [29].

## 3. Oxidative Stress in Animal Models of MI and HF—The Importance of Homocysteine, Vitamin B6, and Folic Acid

Oxidative stress affects the development and progression of clinical and experimental HF. Oxidative stress is defined as an imbalance between the production of reactive oxygen species (ROS) and the endogenous antioxidant defense mechanism. When present in low concentrations, ROS play a significant role in cell homeostasis. Excess of ROS, on the other hand, can cause cell dysfunction and, in some cases, even cell death. Accumulation of ROS in the heart can cause the development and progression in myocardial remodeling, leading to HF [101]. ROS participates in changes of proteins that are responsible for myocardial contractions (calcium “L”-type channels, sodium channels, potassium channels, and sodium potassium channels). In this way, ROS affect the contractility of heart muscle. ROS alter activity of the sarco(endo)plasmatic reticulum Ca^2+^-ATPase (SERCA) and reduce the sensitivity of myofilaments to calcium. Furthermore, ROS induce fibroblast proliferation and activation of heart matrix metalloproteinases, leading to extracellular remodeling [102]. In mice, induced MI increases ROS production, leads to dilatation of the left ventricle, contractile dysfunction, and remodeling. In addition to a drastic increase in oxidative stress, HF is characterized by depletion of the innate antioxidant defense mechanism. In cardiomyocytes, as well as in most cells, the main endogenous components of the antioxidant defense mechanism are superoxide dismutase (SOD), catalase, glutathione peroxidase (GPx), nicotinamide adenine dinucleotide (NAD^+^) and glutathione (GSH). Several studies have noted significant reductions in SOD, catalase, and GPx activity in animal models of HF [103,104,105]. Mice with myocardial injury had reduced activity of SOD and GPx and worse outcomes in comparison to their controls [106,107,108,109,110]. NAD^+^ together with reduced NADH is crucial in initiating the oxidation-reduction reactions responsible for energy production [101]. In addition to its role in the regulation of cellular energy metabolism, NAD^+^ is a precursor for the phosphorylated nucleotide pair NADP^+^/NADPH which plays a leading role in detoxifying ROS. Decreased levels of myocardial NAD^+^ have been found in several animal models of HF. The literature shows significantly reduced activity of nicotinamide mononucleotide adenylyl transferase (Nmnat) in animal models of HF and in patients. This enzyme is responsible for NAD^+^ production [111]. This observation indicates that a decrease in NAD^+^ also occurs in humans. GSH, like NAD^+^, is one of the main antioxidants in mammalian cells. It scavenges radicals and eliminates the products of lipid peroxidation. In an animal model of MI, total GHS was decreased [112,113].

Vitamin B6 is a coenzyme in the glutathione-based antioxidant defense system. Pyridoxal phosphate (PLP) can act as a coenzyme of transsulfuration, allowing the conversion of Hcy to cysteine. Cysteine is involved in the synthesis of reduced glutathione (GSH). In addition to GSH, GPx, glutathione reductase (GR), and glutathione S-transferase (GST) also participate in cellular antioxidant defense [114]. Vitamin B6 deficiency can induce impairment of antioxidative defense mechanism, and thus contribute to oxidative stress. Experimental studies have shown that the antioxidant effect of vitamin B6 is based on the inhibition of xanthine oxidase. Xanthine oxidase and xanthine dehydrogenases represent two faces of oxidoreductase, an enzyme with a significant role in the final stage of purine catabolism and the formation of uric acid and hydrogen peroxide [115]. In animal models of MI, PLP reduced the size of the infarcted lesion and improved cardiac function [116]. As mentioned earlier, vitamin B6 deficiency is associated with HHcy, which is considered to be a risk factor for atherosclerosis. Studies in animal models showed higher level of lipid peroxide in a group of rats deficient in vitamin B6, as well as in a group treated with Hcy, compared to the control group [117]. During oxidative stress, methionine synthase activity is reduced, causing reduced methionine synthesis, while transsulfuration reactions are shifted toward GSH synthesis [118].

Preclinical studies showed that vitamin B6 supplementation could lead to a decrease in Hcy concentration and reduction of oxidative stress, and have a beneficial effect on markers of cardiac function [119].

Folic acid is a free radical scavenger that acts as an antioxidant, protecting the organism from damage caused by accumulation of free radicals. It can act directly or indirectly by competitive inhibition of xanthine oxidase [120]. Its antioxidant role is very important in CVDs [121]. Experimental research has shown that a fat-rich diet in rats causes impairment of cardiac function, but supplementation with folic acid improves left ventricle function [122]. As already mentioned, the folic acid metabolism is related to Hcy metabolism. Intake of a large amount of folic acid increases Hcy remethylation, resulting in a decrease in Hcy level. Folic acid supplementation could therefore decrease Hcy level and cardiovascular risk. Folic acid supplementation can affect posttranslational protein modulation, which can favorably affect the heart contractile function [122]. Studies have shown that folic acid mitigates the oxidative stress induced by HHcy [123]. ROS also damages cell DNA, but folic acid regulates cell growth and DNA repair, especially in HHcy, thus participating in antioxidant protection [124].

Models of HF induced by a single dose of monocrotaline showed that co-application of folic acid and vitamin B6 decreased SOD, GPx activity, and glutathione levels [125,126]. The importance of folate pretreatment in a model of cardiotoxicity caused by doxorubicin showed reduced production of superoxide anions, resulting in a reduction in oxidative stress [127]. Folic acid also increases the coronary flow and nitrite outflow in isolated rat hearts [128] and improves endothelial function in CAD in humans [129,130].

## 4. Inflammation in Animal Models of Myocardial Infarction and Heart Failure—Significance of Homocysteine, Vitamin B6 and Folic Acid

Endothelial dysfunction has a key role in the inflammatory process of atherosclerosis. Vascular inflammation is a consequence of the inability of endothelial cells to counteract ROS, causing increased production of cytokines and smooth muscle cell proliferation. These changes can impair heart function [131].

The regional response after ischemic MI can be divided into four phases [132,133]: (i) necrosis phase, (ii) acute inflammatory phase, (iii) subacute granulation phase, and (iv) chronic scar phase. The necrosis phase occurs immediately after MI cells die and is characterized by necrosis and apoptosis. The acute inflammatory phase corresponds to an inflammatory response that occurs in the first seven days after ischemic MI onset (and the aim of this phase is to absorb necrotic tissue). The subacute granulation phase corresponds to the formation of granulation tissue consisting of proliferated myofibroblasts, which increases the tension strength of the heart muscle, and the proliferation of blood vessels to improve perfusion and enable better cell survival. This phase typically lasts one to three weeks. The chronic scar phase occurs after a month, and is characterized by the formation of fibroblasts, the regression of small blood vessels, and the formation of final scar tissue rich in collagen [134].

The systemic inflammatory response consists of: humoral (cytokines and complement system) and cell-mediated response [134]. The healing process after MI is initiated by TNF-α, IL-6 and IL-1 [135,136,137]. Ischemia and other factors, such as damaged myocytes and ROS, stimulate TNF-α in the acute postinfarction phase. In the early postinfarction phase, a certain level of cytokine production is physiological because it reduces cellular apoptosis. The complementary system stimulates cytokine production (e.g., interlukin-8). These cytokines, together with activated platelet factor (produced in endothelial cells), stimulate the endovascular adhesion of neutrophils [136,137]. These processes increase vascular and tissue inflammation. Excessive regional inflammatory response can have a detrimental effect on the infarct zone, even leading to an increase in the affected zone. Neutrophils, monocytes/macrophages, and mast cells participate in the inflammatory cell-mediated response. Neutrophils from blood vessels migrate to the infarcted zone, where they can reduce local perfusion and lead to an increase of thromboxane B2. As a result, vasoconstriction and platelet aggregation can occur in this area. Monocytes, which become macrophages and secrete various cytokines and growth factors, can also migrate into the damaged heart tissue [134]. Degradation of mast cells leads to the release of fibroblast growth factors, endothelial growth factors, histamine, and other factors that promote fibrosis and angioneogenesis in the myocardium, thus participating in the formation of the final scar tissue [138,139].

C reactive protein (CRP) levels increase after acute MI due to cytokine activation, and it binds to damaged heart muscle cells. This stimulates the cascade of complement components, which can lead to an increase in the size of the infarct zone. In addition, it has proatherogenic and prothrombotic characteristics, so it can be used as a marker of acute MI [134]. The production of IL-6 is stimulated by interferon gamma, IL-1, and TNF-α [140,141]. It affects the inflammatory response and platelet aggregation, and stimulates the proliferation of vascular smooth muscle cells (acts as a procoagulant) [142]. IL-1 is also involved in inflammation after MI. IL-1 receptor type I (IL-1RI) is associated with post-MI inflammation, impaired ventricular function, and scar formation [143,144,145]. IL-18 pretreatment in ischemia/reperfusion models showed significant decreases in infarct size [146].

Vitamin B6 (in the form of PLP) affects the acute inflammation phase after acute MI. It is necessary for the production of cytokines, which are involved in the chronic phase of inflammation, and in the proliferation and activation of lymphocytes [147]. Studies have shown a transient decrease of PLP after acute MI [148,149]. It is known that increasing vitamin B6 levels decrease inflammation, but it has been suggested that a deficiency of vitamin B6 also increases the risk of inflammation or inflammatory diseases [150,151,152]. It has been shown that vitamin B6 lowers CRP [147].

The anti-inflammatory effect of folic acid consists of the reduction of some inflammatory mediators, and it is considered to be a potential drug that has protective effect in CVDs [153]. Decreased folate concentrations can increase inflammation [154]. Another confirmation of the anti-inflammatory effect of folate is that a folate-rich diet reduces the level of IL-6 [155]. Animal models of early atherosclerosis (apoE mice) showed the positive effect of high doses of folic acid on inflammatory status and cholesterol level [156].

In addition to acting as a procoagulant, Hcy in high doses contributes to vascular inflammation. Experiments on ApoE mice showed a 19-fold higher Hcy concentration in the group of mice fed a methionine-rich food (methionine is a substrate for Hcy synthesis) compared to the control group. HHcy was induced in mice with methionine-rich diet, but low folate and vitamins B6 and B12 was associated with increased atherosclerotic lesions in the aortic sinus compared to the control group. The characteristics of these atherosclerotic lesions are an increased number of macrophages and smooth muscle cells. In the same group of mice, expression of vascular cell adhesion molecule/protein 1 (VCAM-1) was 3.7-fold higher [157]. Expression of these molecules is at least partly mediated by activation of NF-κB [158,159], a transcription factor associated with the proinflammatory response [160,161].

## 5. Gasotransmitters in Animal Models of Myocardial Infarction and Heart Failure—Significance of Homocysteine, Vitamin B6 and Folic Acid

Gasotransmitters are a group of regulatory molecules that participate in physiological and pathological functions in mammalian tissues. Nitric oxide (NO), hydrogen sulfide (H_2_S), and carbon monoxide (CO) are involved in many physiological functions [12]. In cardiovascular systems, H_2_S and NO participate in vasorelaxation, stimulation of angiogenesis, and cardioprotection [162,163]. CO participates in relaxation of smooth muscle cells of coronary blood vessels and in cardioprotection [164]. These gasotransmitters have antioxidative potential, by means of which they can fulfill their cardioprotective role [165].

### 5.1. Nitric Oxide

In mammalian tissue, NO is a result of enzymatic and non-enzymatic reactions. The enzyme NO synthase (NOS) produces NO by converting l-arginine to l-citrulline [166]. There are three isoforms of NOS: neural (nNOS), endothelial (eNOS), and inducible (iNOS). The presence of eNOS in the vascular endothelium is particularly important for cardiovascular physiology [12]. Its task is to maintain basal vascular tone by secreting a small amount of NO. NO has been the subject of many studies in ischemic/reperfusion myocardial injury, and eNOS deficiency exacerbates this type of myocardial injury [167]. Along with eNOS, l-arginine reacts with oxygen radicals, which are reduced by NADPH to form NO radicals and l-citrulline. The availability of l-arginine is particularly important for maintaining adequate NO production. The uptake of l-arginine into endothelial cells is enabled by the systemic transporter of y+ cationic amino acids (Cat). Prolonged incubation with a significant concentration of Hcy in bovine aortic endothelial cells leads to a significant dose-dependent decrease in Cat-1 isoform expression [168]. This leads to decreased transmembrane transport of l-arginine, reduced NO production, and suppressed vasodilation after acetylcholine administration [169]. It is not known why Hcy changes l-arginine transport via Cat-1, but it is suggested that oxidative stress induced by Hcy plays an important role, because the function of y+ can be improved with antioxidants [168]. When the amount of l-arginine is small, NO synthesis decreases, and a phenomenon called eNOS “uncoupling” occurs. eNOS is heterodimer comprising of two reductase domains linked to another pair of oxygenase domains. In the reductase domains, electrons from NADPH reduce oxygen with heme as a cofactor. Then the iron-dioxygen complex oxidizes L-arginine to form NO in the oxygenase domain. In the absence of l-arginine, the oxygenase domain, necessary for NO production, is impaired. The reductase domain continues to reduce oxygen, leading to superoxide production. eNOS is thus “uncoupled” [170]. In vitro experiments on Hcy-treated human umbilical vein endothelial cell cultures showed the presence of uncoupled eNOS. The results of this study showed that ROS production was proportional to the activity of eNOS. The results also showed a reduction in the availability of tetrahydrobiopterin (BH4) by 80% [169].

Superoxide from uncoupled eNOS and increased NADPH oxidase reduces residual NO from the still-functional eNOS. The product of this reaction is peroxynitrite. Peroxynitrite is an oxidizing agent that oxidizes and decreases the level of BH4. Progressive reduction of BH4 due to peroxynitrite production may trigger a cycle in which eNOS uncoupling is further impaired. This increases BH4 destruction [171]. Another mechanism by which Hcy impairs the endothelial function and dilation is through the asymmetric dimethylarginine (ADMA) molecule pathway. l-arginine incorporated into proteins goes through methylation, then, with the help of the enzyme protein-arginine methyltransferase, ADMA is synthesized during the posttranslational protein change [172]. Methylated protein hydrolyzed during protein turnover releases ADMA. Due to its structural similarity to L-arginine, ADMA competes with L-arginine to inhibit eNOS [173]. The main pathway of ADMA metabolism is the production of citrulline methylamine, a reaction catalyzed by dimethylarginine dimethylaminohydrolase (DDAH). A small fraction of ADMA is metabolized to α-keto acids or excreted by kidneys.

Hcy can influence DDAH activity, thus preventing ADMA metabolism, resulting in a reduced amount of NO [174]. During ischemia, pH in tissue decreases, and the oxygen-dependent activity of NOS becomes limited, which makes the production of NO by NOS-independent enzymatic and non-enzymatic reduction of endogenously created nitrites or nitrates particularly significant [12]. In myocardial ischemia, NO is a potent vasodilator that allows perfusion of damaged myocardium [175]. NO reversibly inhibits respiration in mitochondria, which allows better cellular oxygenation in tissues distant from blood vessels [176,177]. NO is also important because it inhibits the adhesion of neutrophil to the endothelium of blood vessels. This is important, because neutrophilic adhesion stimulates the activation of other leukocytes and the production of superoxide radicals, which further damage the endothelium and myocardium [178,179]. NO also has an antiaggregating role [180], and it inhibits apoptosis [181].

Nitrites and NO alleviate myocardial damage. For this reason, donors of nitrites and NO could be potential agents for the reduction of infarcted lesions. In this context, sodium nitrite was effective in experimental models of MI. Its effects were evident when it was applied before the occlusion, but also when it was applied as a peri-conditioning agent [182,183,184]. Studies on the Langendorff isolated and perfused heart model showed that nitrite infusion before ischemia reduced the infarct lesion and induced a better recovery of left ventricular function [185]. A study using ischemia/reperfusion models in mice was performed to investigate nitrite’s cardioprotective role. In this study, left coronary artery occlusion caused ischemia for 30 minutes, and subsequent reperfusion lasted for 24 h. Sodium nitrite was applied during myocardial ischemia, and exhibited protective effects on cardiomyocyte necrosis [186]. Data from the literature show that low-molecular-weight thiol compounds react with NO to form s-nitrosothiols (RSNOs), which have similar activity to NO.

Normal vascular endothelial cells can alter the side effects of Hcy by increasing NO production and promoting the production of S-nitrosohomocysteine (SNOHcy). In this way, endothelial cells protect the blood vessel wall from the harmful effects of Hcy. Prolonged exposure of endothelial cells to Hcy can disrupt NO response. Disruption can happen due to reduced NO production by eNOS or to reduced bioavailability of NO (through the formation of SNOHcy from Hcy and NO). Experimental studies in rats showed that elevated levels of Hcy can significantly mitigate the systemic NO-mediated vasorelaxation induced by nitric oxide donors. SNOHcy, S-nitrocysteine (SNOCys) and sodium nitroprusside (SNP) function as NO donors. They all have similar hypotensive effects, which can be similarly impaired in HHcy. SNOHyc releases NO (SNOHyc → Hcy + NO) to achieve a vasodilatory effect. If Hcy is in excess (Hcy infusion), the reaction moves in the direction of SNOHyc production (Hcy + NO → SNOHyc) and not in the direction of NO release. This is the reason the bioavailability of NO is lower, leading to less efficient vasodilatation [187].

Hcy reduces the activity of intracellular glutathione peroxidase [188]. Decreased glutathione peroxidase activity can make NO more susceptible to oxidative inactivation, which in turn reduces NO bioavailability. These two pathways can be complementary [187]. As mentioned earlier, Hcy acts as a procoagulant. HHcy is responsible for accelerated platelet activation, accelerated coagulation, and decreased fibrinolysis, all of which lead to posttranslational changes in fibrin fibers [189]. In preclinical experiments, folic acid treatment increased plasma folate concentrations and suppressed thrombosis in mice deficient in apolipoprotein E and LDL receptors [190]. A double-blind study including 158 healthy siblings of patients with homocysteinemia with premature atherothrombotic disease demonstrated a connection between folic acid and vitamin B6 administration and reduced incidence of pathological changes in electrocardiographic stress tests [191]. The exact mechanism for the development of accelerated thrombosis has not been elucidated, but it is suggested that reduced NO bioavailability, which stimulates platelet activation, is the main reason [192].

### 5.2. Hydrogen Sylfide

**Figure 2:**
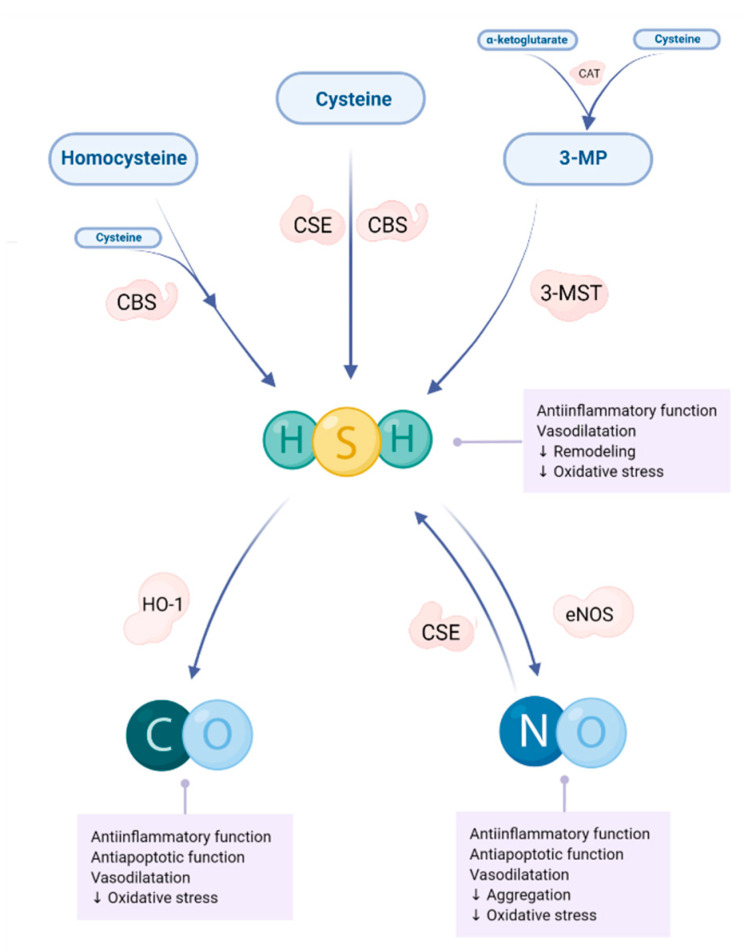
Relationship between H_2_S, NO and CO, and their roles in cardioprotection. CAT—cysteine aminotransferase, CBS—cystathionine β-synthase, CSE—cystathionine γ-lyase, CO—carbon monoxide, eNOS—endothelial nitric oxide synthase, HO-1—heme oxygenase-1, H_2_S—hydrogen sulfide, NO—nitric oxide, 3-MP—3-mercaptopyruvate, 3-MST—3-mercaptopyruvate sulfurtransferase. Substrates are represented in the oval boxes and enzymes in the bubbles next to the line pointing to the product. Arrow pointing up (↑) represents increased process or function, and arrow pointing down (↓) represents decreased process or function.

H_2_S results from endogenous sources (cysteine) found in blood and other tissues. H_2_S is a product of cysteine in the process of desulfurization. The production of H_2_S is catalyzed by the same enzymes, i.e., CBS and CSE, and 3-mercaptopyruvate sulfurtransferase (3-MST), involved in transsulfuration. H_2_S can be synthesized from cystationine by CSE. H_2_S can also be produced by CBS from cysteine. This cysteine is created from cysthationine (formed in the reaction of serine and Hcy). After the cysteine aminotransferase (CAT) forms 3-mercaptopyruvate (3MP), the 3MP can be broken down with 3-MST to form H_2_S. There is a lot of CSE in the heart and little CBS; thus, the heart is considered to be a significant source of H_2_S production [12]. Changes in H_2_S metabolism can lead to hypertension, diabetes, cirrhosis, neurodegenerative diseases, asthma, erectile dysfunction, atherosclerosis, HF, inflammation, and sepsis [193,194]. H_2_S in the cardiovascular system has a cardioprotective role; it affects vasodilation and the reduction of blood pressure. There is a strong bond between H_2_S and NO. This is based on ability of H_2_S to activate eNOS and increase the amount of NO [14]. NO can increase H_2_S biosynthesis by CSE activation [195]. Figure 2 shows the crosslink of these gasotransmitters, and their effects on the cardiovascular system. H_2_S also plays a significant role in myocardial fibrosis, which can cause the development of HF. It also inhibits the TGF-β1/Smad3 signaling pathway, reducing collagen deposition in the myocardium [196].

H_2_S, like many other molecules, can have beneficial effects by acting through various pathways, but the main one is associated with oxidative stress and NO [197]. Ischemia/reperfusion injury of the myocardium leads to tissue destruction and HF. Although reperfusion improves ischemia, it also leads to changes such as inflammation and oxidative stress [198]. An in vivo ischemia/reperfusion model in mice showed that H_2_S acts in favor of cardio protection by means of inhibiting inflammation, reducing the size of the infarcted zone, and preserving the structure and function of the left ventricle. This experiment showed that the modulation of the endogenous production of H_2_S by overexpressing cardiac CSL leads to a significant reduction of myocardial injury. This suggests that the administration of H_2_S or its increased endogenous production may be significant for clinical use in ischemic disorders [199]. The mechanisms of H_2_S cardioprotection in ischemia/reperfusion injury include the maintenance of mitochondrial function, the reduction of apoptosis in heart muscle cells, anti-inflammatory response, and antioxidant effects. Mitochondria play a role in energy production and cell survival. After MI, they maintain oxidative phosphorylation, and thus protect the cells. In vitro experiments showed reduced oxygen consumption after treatment with H_2_S [199]. During reperfusion, H_2_S maintains mitochondrial function by increasing the efficiency of complexes I and II in the electron transport chain.

In ischemia, ROS can impair mitochondrial function, which may exacerbate myocardial damage [200]. High doses of this gasotransmitter can inhibit cytochrome C oxidase, and thus reduce cellular respiration, leading the cell to a state of reduced metabolism, thereby protecting the cell [201]. This inhibition of respiration limits ROS production, protecting the myocardium from ischemia/reperfusion injury [200]. H_2_S has an antioxidant effect via the Nrf-2 signaling pathway. Nrf-2 can be translocated from cytosol to the nucleus, and it can induce various antioxidant proteins there. It stimulates antioxidant protection and reduces oxidative stress. Studies have shown that diallyl trisulfide (DATS), a donor of H_2_S, after MI, allows the translocation of Nrf-2 from the cytosol into the nucleus, maintaining unchanged its total amount in the cell [202]. Activated Nrf-2 can activate detoxification genes in the nucleus. This leads to activation of heme oxygenase 1 (HO-1), superoxide dismutase, and catalase. H_2_S also increases glutathione levels and reduces oxidative stress. Apart from the fact that H_2_S itself has an antioxidant effect, it can also act together with NO, because it activates eNOS and increases the bioavailability of NO. H_2_S can prevent inflammation, because it prevents the adhesion of leukocytes to the wall of the blood vessels and inhibits the adhesion and expression of adhesion molecules. It also slows down the remodeling of the heart and promotes angiogenesis in congestive HF. Angiogenesis involves the remodeling of the extracellular matrix and its incorporation into capillaries [200]. HHcy affects the synthesis of H_2_S. Under physiological conditions, the concentration of total cysteine in plasma is higher than the concentration of Hcy. Cysteine is the source of 70% of H_2_S. High Hcy concentration overpowers cysteine, and Hcy becomes a key source of H_2_S. It has been shown that Hcy can reduce H_2_S production [203]. Lower H_2_S levels were found in mice with HHcy [204] and in mice with intracerebrally administered Hcy [205].

### 5.3. Carbon Monoxide

Endogenous CO is formed by the degradation of heme in the reaction catalyzed by heme oxygenases subtypes 1 and 2 (HO-1, HO-2). This reaction produces iron ion (Fe^2+^), biliverdin and CO. Biliverdin rapidly converts to bilirubin in presence of oxygen and NADPH. This reaction is significant for bile and iron metabolism. In cardiomyocytes, the expression of HO-1 and the HO-2 is significant. HO-1 expression is inducible, while HO-2 is constitutive. Various stressful conditions, including MI, can lead to increased HO-1 expression. Induction of HO-1 is particularly strong in ischemia/reperfusion models. In these models, HO-1 is increased in coronary arteries, which contributes to the maintenance of perfusion via vasodilatation [206]. In addition to this effect, CO shows anti-apoptotic, antihypertensive and anti-inflammatory effects (Figure 2) [164,206,207,208]. At low concentrations, CO inhibits the expression of proinflammatory cytokines TNF-α, IL-1β, macrophage inflammatory protein-1β (MIP-1β), and increases the expression of anti-inflammatory cytokines, such as IL-10 [209].

Various CO-releasing molecules (CORMs) reduce the size of the infarcted lesion without affecting arterial blood pressure, heart rate, and carboxyhemoglobin concentration [71]. In a model of MI induced by ligation of the left descending artery in mice, CO-releasing molecules improve left ventricular remodeling [210]. Pretreatment with CORM 24 to 72 h before occlusion in mice reduced the size of the infarcted zone [211]. Synthetic CO-releasing molecules have beneficial effects on the structural and functional recovery of the myocardium in models of left descending coronary artery ligation in rats. They also increase the number of cardiomyocytes in damaged areas, as well as blood vessels [212]. CO and NO have similar effects (Figure 2), both acting as signaling molecules and messengers. CO, as a signaling molecule, has prolonged effects on the cardiovascular system compared to NO and H_2_S [213]. H_2_S can induce activity of HO-1 and increase CO production [214]. The effects of CO on the cardiovascular system include: modulation of autonomic nervous system activity on pacemaker cells and other muscle cells of the heart, vasodilation, changes in heart rate and contraction strength [213]. CO alters the electrophysiological activity of the heart either by its direct action or by its effects on the signaling pathways in the cell. The level of CO also changes the amount of NO by nitrosylation of ion channels and ion transporters, or directly by specific signaling molecules. NO/S nitrosylation increases K^+^ current, which creates a resting potential in the heart muscle. It can also affect the turnover of Na^+^/K^+^ ATPase in the hypoxic heart, and cause the release of Ca^2+^ from the sarcoplasmic reticulum via the ryanodine receptor complex [213]. Electrophysiological and contractile changes affected by CO are caused by an increase in the late Na^+^ current in the heart. NO is a messenger in this process [215,216]. CO acts on soluble guanylyl cyclase, by binding it and increasing the amount of cGMP. This means of forming cGMP is controversial, because the activation of soluble guanylyl cyclase caused by CO induction is lower than that caused by NO induction. In cases where NO is present, the interaction of guanylyl cyclase with CO results in a small amount of cGMP. Some endogenous substances can increase the sensitivity of soluble guanylyl cyclase to CO [217]. The literature shows that CO inhibits CBS, an enzyme involved in H_2_S production. CO-dependent inhibition of CBS can influence the remethylation cycle, and is associated metabolic pathways for methionine maintenance and polyamine production [218].

## 6. Conclusions

Vitamin B6 and folic acid deficiency can cause Hhcy, which is associated with myocardial injury. Models of MI and HF include complex processes: oxidative stress, endothelial dysfunction, gasotransmitter activity, inflammation, and extracellular fibrous changes. The supplementation of vitamin B6 and folic acid in experimental models of MI and HF could improve heart function. Treatment with vitamin B6 and folic acid could improve vasodilatation, coronary flow, reduce oxidative stress and inflammation.

## Data Availability

Not applicable.

## Abbreviations

3MP	3-mercaptopyruvate
3-MST	3-mercaptopyruvate suphurtrasferase
5-MTHF	5-methyltetrahydrofolate
5′NT	Nucleotidase
ADA	Adenosine deaminase
ADMA	Asymetric dimethylarginine
AdoMet	S-adenosyl methionine
ALP	Alkaline phosphatase
AMI	Acute myocardial infarction
AST	Aspartate aminotransferase
BH4	Tetrahydrobiopterin
BHMT	Betaine homocysteine methyltransferase
BNP	B-type natriuretic peptide
CAD	Coronary artery disease
Cbl	Cobalamin
Cat	Cationic amino acids
CAT	Cysteine aminotransferase
CBS	Cystathionine β-syntase
CK	Creatine kinase
CO	Cabon monoxide
CORM	CO-releasing molecules
CRP	C-reactive protein
CSE	Cystathionine γ-lyase
DALYs	Disability-adjusted life years
DATS	Diallyl trisulfide
DDAH	Dimethylarginine dimethylaminohydrolase
DHF	Dihydrofolate
DHFR	Dihydrofolate reductase
DMG	Dimethylglycine
dTMP	Deoxythymidine monophosphate
dUMP	Deoxyuridine mnonophosphate
DVT	Deep venous thrombosis
eNOS	Endothelial NOS
Gal-3	Galectin-3
GC	Guanylate cyclase
GDF-15	Growth differentiation factor-15
GGT	Gamma-glutamyl transferase
GPx	Glutathione peroxidase
GR	Glutathione reductase
GSH	Reduced glutathione
GSSG	Oxidized glutathione
GST	Glutathion S-transferase
H_2_S	Hydrogen sulphide
Hcy	Homocysteine
HDL	High density lipoprotein
HF	Heart failure
H-FABP	Heart fatty acid-binding protein
HHcy	Hyperhomocysteinemia
HO	Haem oxygenase
hs-cTN	High sensitive cardiac troponin
hs-Tn	High sensitive troponin
HTL	Homocystein thiolactone
IL-1	Inerleukin-1
IL-1β	Interleukin-1β
IL-18	Interleukin-18
IL-6	Interleukin-6
iNOS	Inducibile NOS
IP_3_	Inositol triphosphate
ISO	Isoprenaline
LAD	Left anterior descending
LCA	Left coronary artery
LDH	Lactate dehydrogenase
LDL	Low density lipoprotein
LDLR	Low density lipoprotein receptor
Lp-PLA A2	Phospholipase A2 associated lipoprotein
LVEDV	Left venticle end-diastolic volume
LVEF	Left ventricular ejection fraction
MAPK	Mitogen-activated protein kinases
MAT	Methionine adenosyltransferase
MDA	Malondialdehyde
MetRS	Methionyl-tRNA synthetase
MI	Myocardial infarction
MIP-1β	Macrophage inflammatory protein-1β
MMP-2	Matrix metalloproteinase-2
MMP-9	Matrix metalloproteinase-9
MMPs	Matrix metalloproteinases
MS	Methionine syntase
MSR	Methionine syntase reductase)
MYO	Myoglobin
NAD^+^	Nicotinamide adenine dinucleotide
NF-κB	Nuclear factor kappa B
NMDA	N-methyl-D-aspartate
Nmnat	Nicotinamide mononucleotide adenylyl transferase
nNOS	Neuronal NOS
NO	Nitric oxide
NOS	Nitric oxide synthase
NS	Nucleosidase
NT-proBNP	N-terminal prohormone B-type natriuretic peptide
PKA	Protein kinase type A
PKC	Protein kinase type C
PLP	Pyridoxal phosphate
PON 1	Paraoxonase 1
PTX-3	Petraxin
RAAS	Renin-angiotensin-aldosterone system
ROS	Reactive oxygen species
RSNOs	S-nitrosothiols
SAHH	S-adenosyl homocysteine hydrolase
SAM	S-adenosyl methionine
sCD40L	Soluble ligand CD40
SERCA2a	Sarcoplasmatic reticulum atpase 2a
SHMT	Serine hydroxymethyltransferase
SNOCys	S-nitrocysteine
SNOHyc	S-nitrosohomocysteine
SNP	Sodium nitroprusside
SNS	Sympathetic nervous system
SOD	Superoxide dismutase
THF	Tetrahydrofolate
TNFR 1	TNF-α receptor-1
TNFR 2	TNF-α receptor-2
TNF-α	Tumor necrosis factor-α
TYMS	Thymidylate syntase
VCAM-1	Vascular cell adhesion molecule/protein 1
VLDL	Very low-density lipoprotein
vWF	Von Willebrand factor
ECG	Electrocardiography
XO	Xanthine oxidase
